# Role of Cx43 in iPSC-CM Damage Induced by Microwave Radiation

**DOI:** 10.3390/ijms241612533

**Published:** 2023-08-08

**Authors:** Yue Yin, Xinping Xu, Dayan Li, Binwei Yao, Haoyu Wang, Li Zhao, Hui Wang, Ji Dong, Jing Zhang, Ruiyun Peng

**Affiliations:** Beijing Institute of Radiation Medicine, Beijing 100850, China

**Keywords:** microwave, iPSC-CM, Cx43, intercalated disc, autophagosome-like bodies, electrical conduction

## Abstract

The heart is one of the major organs affected by microwave radiation, and these effects have been extensively studied. Previous studies have shown that microwave-radiation-induced heart injury might be related to the abnormal expression and distribution of Cx43. In order to make the research model closer to humans, we used iPSC-CMs as the cell injury model to investigate the biological effect and mechanism of iPSC-CM injury after microwave radiation. To model the damage, iPSC-CMs were separated into four groups and exposed to single or composite S-band (2.856 GHz) and X-band (9.375 GHz) microwave radiation sources with an average power density of 30 mW/cm^2^. After that, FCM was used to detect cell activity, and ELISA was used to detect the contents of myocardial enzymes and injury markers in the culture medium, and it was discovered that cell activity decreased and the contents increased after radiation. TEM and SEM showed that the ultrastructure of the cell membrane, mitochondria, and ID was damaged. Mitochondrial function was aberrant, and glycolytic capacity decreased after exposure. The electrical conduction function of iPSC-CM was abnormal; the conduction velocity was decreased, and the pulsation amplitude was reduced. Wb, qRT-PCR, and IF detections showed that the expression of Cx43 was decreased and the distribution of Cx43 at the gap junction was disordered. Single or composite exposure to S- and X-band microwave radiation caused damage to the structure and function of iPSC-CMs, primarily affecting the cell membrane, mitochondria, and ID. The composite exposure group was more severely harmed than the single exposure group. These abnormalities in structure and function were related to the decreased expression and disordered distribution of Cx43.

## 1. Introduction

With the rapid development of technology, human beings are exposed to microwave radiation from industry [1,2], medical treatment [3], communications [4], and other equipment every day. The communication equipment represented by the 5G network and the radar widely used in the military field uses S- and X-band microwaves. Previous studies have shown that while microwave technology brings great convenience to social development, long-term exposure to large doses of electromagnetic radiation will cause damage to multiple organ systems of organisms to varying degrees [5,6]. However, there is a lack of relevant studies on the biological effects and damage mechanisms of S- and X-band microwave radiation, and pioneering studies on the heart are rare. Therefore, we set out to study the effects of S- and X-band microwave radiation on the heart and the possible injury involved.

In previous studies, H9C2 and primary rat cardiomyocytes were selected as the research models for the biological effects of microwave radiation [7]. Due to the limitations of the selection of subjects and the lack of direct data connections from animals to humans, it was difficult to translate the research results to the human condition. In recent years, with the rapid development of regenerative medicine, iPSC-CMs have made a new breakthrough in the treatment of clinical heart diseases [8,9]. Therefore, we decided to use this cell type to establish in vitro models of cellular damage and study the effects and mechanisms involved in microwave-radiation-induced cardiac injury.

It is well known that the heart has unique biological characteristics, including the unique intercalated disc structure and an abundance of mitochondria. The intercalated disc is the structural basis for maintaining electrical and chemical conductivity between cardiomyocytes. The gap junction is an important part of the intercalated disc, and it is one of the key factors affecting action potential propagation and electrical coupling between cardiomyocytes [10]. Our research team has previously found that microwave radiation could cause electrocardiogram abnormalities, Ca^2+^ content disturbance, and mitochondrial cavitation swelling in rats, and these changes might suggest that cardiac electrophysiology and energy transfer are impeded, but there is currently no direct evidence that links these findings to damage on the cellular level [7,11,12].

In this study, disturbances in electrophysiological properties and cellular energy metabolism served as readouts of cardiac injury after microwave radiation. Cx43, the most important connexin in the heart [13], has an important role in the transmission of chemical and electrical signals, and its location at the cell ends is a crucial determinant of its function. Therefore, we analyzed whether the expression and distribution of Cx43, and especially its end-to-end connection, change after radiation. To gain further insight into the mechanism of cellular radiation damage, we analyzed the electrophysiology, mitochondrial function, and ultrastructure of cardiomyocytes, attempting to devise new future treatments for cardiac injury caused by microwave radiation.

## 2. Results

### 2.1. Identification of Cell Differentiation

iPSC-CMs are a cell model that so far has not been employed to study the biological effects of microwave radiation. Before moving on to the microwave radiation tests in this work, the cell type and differentiation state of iPSC-CMs were first identified. Myocardial markers TNNT2/cTNT and Nkx2-5 genes were used for immunofluorescence staining. NKx2-5 indicated early cardiac mesoderm, and the positive signal increased first and then decreased with the increase in cell differentiation days. TNNT2/cTNT is a myocardial-specific marker that is consistently and stably expressed in the myocardium [14].

**Figure 1:**
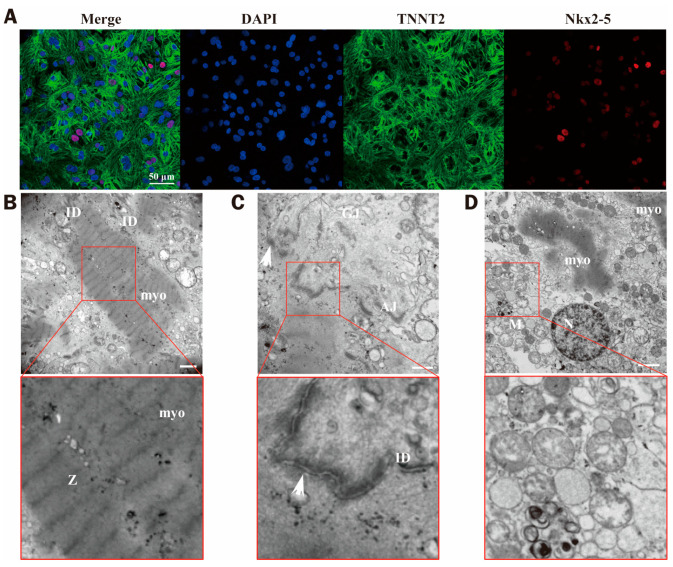
Identifications of iPSC-CMs. (**A**) Cell differentiation identification. The immunofluorescence images of cells on the 7th d after recovery, DAPI (blue), TNNT2 (green), Nkx2-5 (red), scale bar = 50 μm. (**B**–**D**) Ultrastructure of iPSC-CMs. (**B**) scale bar = 500 nm, (**C**) scale bar = 500 nm, (**D**) scale bar = 2 μm (myo: myocardium, Z: Z-line; N: nucleus, ID: intercalated disc, M: mitochondria, GJ: gap junction, AJ: adherence junction, the arrow points to desmosomes).

The cells developed consistently on the dish’s bottom, as shown in Figure 1A. After recovery, TNNT2/cTNT was persistently expressed in the cells, and the Nkx2-5-positive cells were evenly distributed. The ultrastructure of the cells was clearly characterized by myocardial fibers, and the Z-lines were neatly arranged. Desmosomes, tight junctions, and gap junction structures were all visible in the intercalated disc structure, and the cells also contained a large number of mitochondria (Figure 1B–D).

Based on Nkx2-5- and TNNT2/cTNT-specific staining and ultrastructure, the cells were shown to be cardiomyocyte-like.

### 2.2. Cell Membrane Damage after Microwave Radiation

#### 2.2.1. Cell Activity Decreased and Membrane Permeability Increased

**Figure 2:**
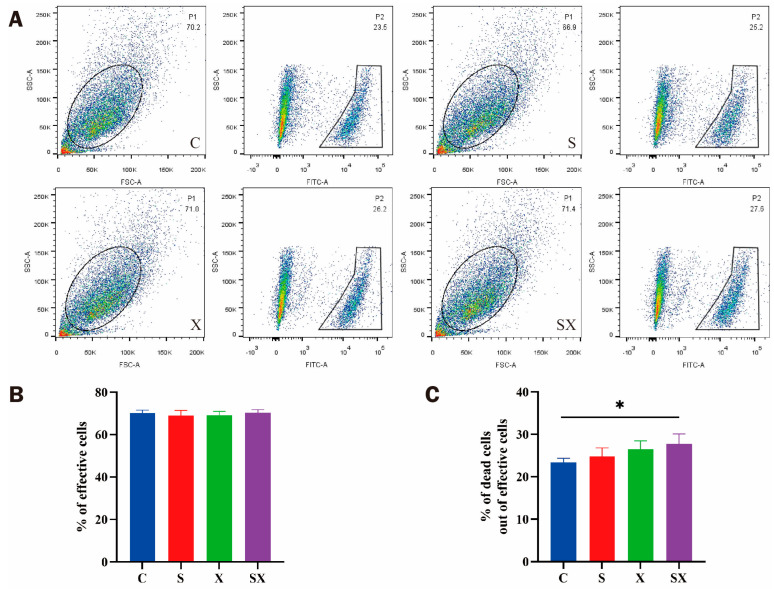
Viability of iPSC-CM membrane changed after microwave exposure. (**A**) Results of iPSC-CM viability analyzed using flowjo. (**B**) Percentage of effective cells. (**C**) Percentage of dead cells, * *p* < 0.05.

Previous studies have shown that apoptosis occurs in cells after microwave radiation [15,16]. In order to detect cell activity after microwave radiation, a Live/Dead kit was used to analyze the fluorescence intensity of live cells and dead cells according to the difference in cell membrane permeability (Figure 2A). The reactive dye can permeate the compromised membranes of necrotic cells and react with free amines both in the interior and on the cell surface, resulting in intense fluorescent staining. In contrast, only the cell-surface amines of viable cells are available to react with the dye, resulting in relatively dim staining.

One hour after exposure, the viability of iPSC-CMs was decreased, the number of dead cells in the radiation groups was higher than that in the C group, and the percentage of dead cells in the SX group was the highest (*p* < 0.05, Figure 2B,C).

#### 2.2.2. The Contents of Myocardial Enzymes and Injury Markers in iPSC-CMs Increased

**Figure 3:**
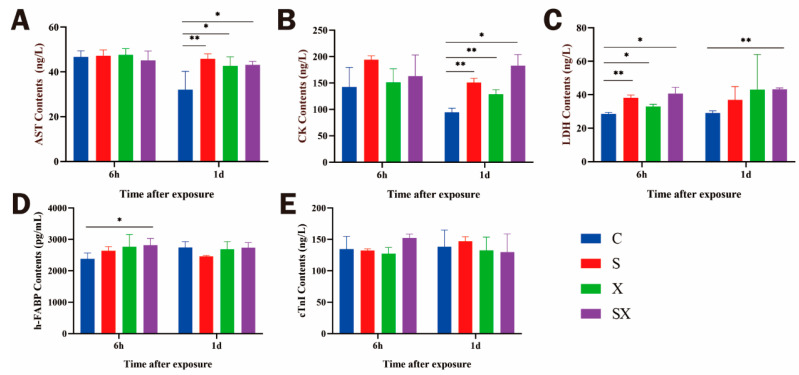
Changes in myocardial enzymes and injury markers in iPSC-CMs after microwave exposure. Contents of AST (**A**), CK (**B**), LDH (**C**), h-FABP (**D**), and cTnI (**E**) as determined by ELISA assay in cell culture medium. * *p* < 0.05, ** *p* < 0.01.

**Figure 4:**
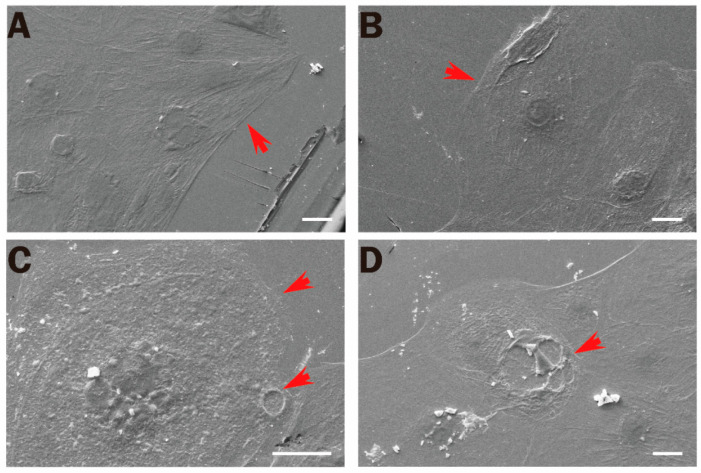
Changes in structure of iPSC-CM membrane after microwave exposure depicted by SEM. (**A**–**D**) Group C (control), group S, group X, and group SX, respectively; scale bar = 20 μm; red arrows emphasize the changes.

As shown in Figure 4C, compared with the C group, the contents of LDH in the radiation groups were significantly increased (*p* < 0.01 or 0.05) at 6 h after exposure; the contents of AST, CK, and LDH in the radiation groups were significantly increased (*p* < 0.01 or 0.05, Figure 3A–C) at 1 d after exposure. The content of h-FABP in the SX group increased 6 h after exposure (*p* < 0.05, Figure 3D). The non-significant change in cTnI might be related to different injury degrees and troponin subunits (*p* < 0.05, Figure 3E). After radiation, the content level of myocardial enzymes increased, which might be related to the damage to the cell membrane.

#### 2.2.3. Membrane Ultrastructure Damage in iPSC-CMs

On the 1st d after exposure, the membrane ultrastructure of iPSC-CMs was observed using SEM. In the C group, cells were polygonal, with smooth cell edges, tight intercellular connections, fewer folds on the cell membrane surface, and complete cell morphology (Figure 4A). In the S group, cells showed that the edges were unsmooth, the surface of the cell membrane was less wrinkled, and the cell was complete (Figure 4B). The surface of the cell membrane in the X group was uneven (Figure 4C). The surface of the cell membrane in the SX group was rough, with more folds; membrane rupture was observed (Figure 4D). Microwave radiation could change the morphology of the cell membrane and even rupture the membrane, which might induce an increase in myocardial enzyme activity.

### 2.3. Energy Metabolism Disorder of iPSC-CMs after Microwave Radiation

#### 2.3.1. Abnormal Mitochondrial Ultrastructure

**Figure 5:**
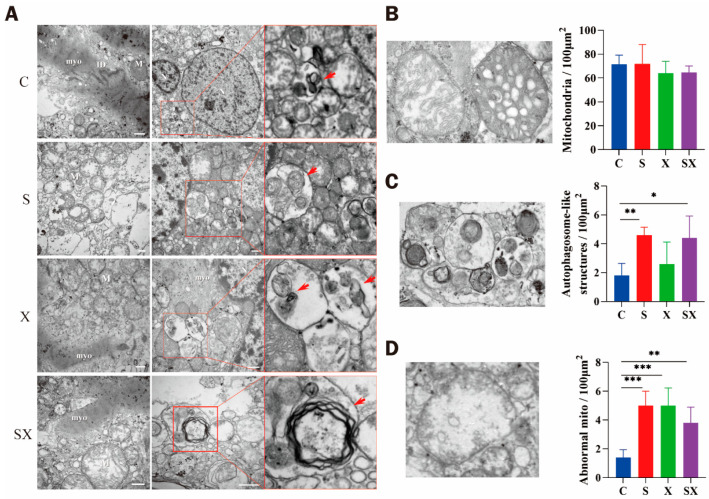
Abnormal mitochondrial structure after microwave exposure imaged using TEM. (**A**) Abnormal ultrastructure of mitochondria, scale bar = 500 nm (myo: myocardium, N: nucleus, ID: intercalated disc, M: mitochondria; the red arrowheads point to the myelin-like structures). (**B**) Mitochondria with normal structures. (**C**) Mitochondria with autophagosome-like structures. (**D**) Mitochondria with abnormal structures. (**B**–**D**, right) Statistics of the numbers of three types of mitochondria per 100 μm^2^ view after exposure. * *p* < 0.05, ** *p* < 0.01, *** *p* < 0.001.

One day after exposure, the cells of the C group showed that the nucleus was completely oval, showing abundant mitochondria and occasionally a mitochondrial myelin-like structure; the intercalated disc structure was clear and complete, in the shape of a broken line; and the Z-line of myocardial fibers was neatly arranged. In the S group, the mitochondrial structure was incomplete, and the mitochondrial cristae were damaged. In the X group, myocardial fibers were sparse, the structure of the nucleus was abnormal, and the structure of the intercalated disc was incomplete. In the SX group, the Z-lines of myocardial fibers were blurred (Figure 5A). In these images of each group, myelin-like structures could be observed in mitochondria, suggesting that they might be related to autophagy.

According to the observation of cell ultrastructure at 1 d after radiation, we found that mitochondrial damage was the most severe. In addition to cavitation of mitochondrial cristae and mitochondrial swelling, the presence of autophagosome-like bodies was discovered in mitochondria and appeared as myelin-like structures. To assess the extent of mitochondrial damage, we divided mitochondria into three categories, namely structurally normal mitochondria (Figure 5B), structurally abnormal mitochondria (Figure 5C), and mitochondria with autophagosome-like structures (Figure 5D), and counted the number of each type of mitochondria.

There was no difference in the total number of mitochondria among the groups at 1 d after exposure (*p* > 0.05), and there were both lamellar cristae mitochondria and tubular cristae mitochondria. Abnormal mitochondria were significantly increased in the radiation groups (*p* < 0.01 or 0.001), and myelin-like structures were significantly increased in the S and SX groups (*p* < 0.05 or 0.01).

The ultrastructural damage in iPSC-CMs, especially in mitochondria, was manifested as an increase in abnormal mitochondria with defective mitochondrial cristae and autophagosome-like structures. It was speculated that the formation of a large number of myelin-like structures might be related to autophagy caused by mitochondrial damage after exposure [17].

#### 2.3.2. Reduced Mitochondrial Respiration Capacity

Because the heart is rich in mitochondria, the above ultrastructural results showed that iPSC-CMs also had abundant mitochondria and abnormal mitochondria after radiation. Since it was speculated that the mitochondria were damaged after microwave exposure as described earlier, we selected the time point 1 h after exposure to detect the mitochondrial function of cells. The OCR of cells at different stages after dosing was measured by sequentially adding targeted drugs to the ETC, and key parameters reflecting mitochondrial function were obtained, including basal respiration, proton leak, maximal respiration, ATP production, and spare respiratory capacity [18].

**Figure 6:**
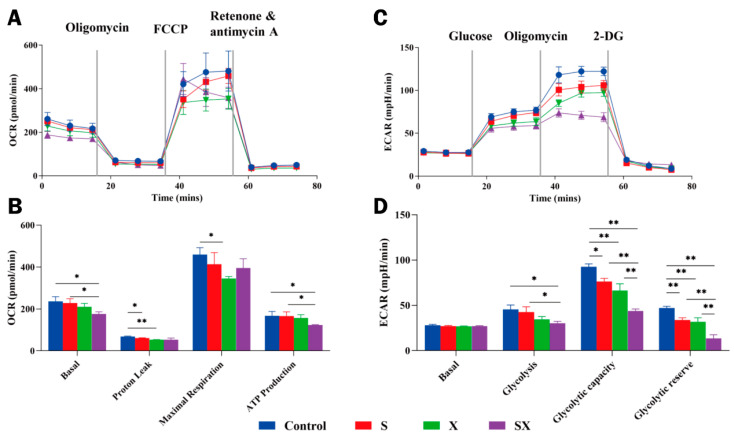
Dysfunction of mitochondria and disturbance of energy metabolism after microwave exposure. (**A**) Mitochondrial respiration and (**B**) statistical analysis of OCR. (**C**) Glycolytic capacity and (**D**) statistical analysis of ECAR. * *p* < 0.05, ** *p* < 0.01.

The results showed that compared with the C group, the basal respiration of the SX group was affected after microwave exposure; that is, the energy demand of the cells was reduced under basal conditions (*p* < 0.05). After adding oligomycin to inhibit ATP synthase, proton leak was significantly decreased in the S and X groups (*p* < 0.05 or 0.01), indicating that mitochondrial damage and the ability of cells to regulate mitochondrial ATP production were reduced. After adding FCCP for uncoupling, the maximum respiration rate in the X group was significantly reduced (*p* < 0.05), indicating that the maximum respiration of cells was impaired; the rate of ATP production in the SX group was significantly decreased (*p* < 0.05), indicating that ATP production could not meet the energy needs of cells. The basal respiration rate and ATP production rate in the SX group were significantly lower than those in the S group (*p* < 0.05). After microwave exposure, the mitochondria of the cells in the radiation group were damaged, which affected the normal energy metabolism of the cells, resulting in a decrease in the production of ATP, thus affecting the normal physiological processes of the cells (Figure 6A,B).

#### 2.3.3. Abnormal Glycolytic Capacity

Glycolysis and oxidative phosphorylation are two key energy production pathways in cells. The most important physiological significance of anaerobic oxidation of glucose is the rapid supply of energy, which is important for myocardial contraction. The content of ATP in the myocardium is so low that it could be used up within a few seconds of myocardial contraction. Even if oxygen is not deficient, it is too late to meet the demand because the reaction process of aerobic oxidation of glucose is too long. However, ATP could be quickly obtained through glycolysis to supply energy to the myocardium [19,20].

This study found that the basal metabolism of the C group and that of the radiation groups were the same. The glycolytic capacity of the radiation groups decreased (*p* < 0.05), the maximum capacity of cells to utilize glycolysis decreased (*p* < 0.05 or 0.01), and the glycolytic reserve capacity also decreased (*p* < 0.01). The damage was dose-dependent, and the combined exposure was more severe than the single exposure (Figure 6C,D).

After microwave exposure, oxidative phosphorylation and glycolysis were damaged to some extent, resulting in abnormal energy metabolism in iPSC-CMs.

### 2.4. Conduction Damage in iPSC-CMs after Microwave Radiation

#### 2.4.1. Ultrastructure of ID

Cardiomyocytes are connected with each other through IDs, forming a complex 3D network structure that makes the ventricular muscle form a whole. IDs are the structural units of mechanical connection and electrical conduction between cardiomyocytes. IDs consist of three special junctions: gap junctions for electrical conduction and desmosomes and adherence junctions for mechanical coupling [21]. Studies have found that when certain physical factors (such as infrasound or electromagnetic pulses) act on the heart, the Cx43 expression changes, the gap between IDs widens and electrical conduction becomes dysfunctional [22,23].

**Figure 7:**
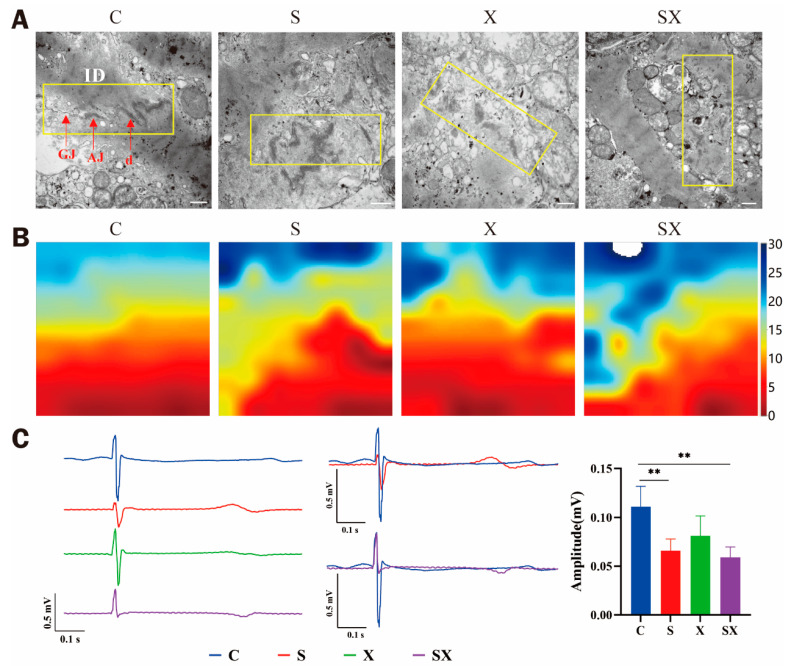
Structural damage in IDs and electrical conduction dysfunction after microwave exposure. (**A**) Abnormal ultrastructure of IDs, scale bar = 500 nm. The yellow boxes show the IDs. (**B**) Electrical mapping recording of the iPSC-CMs. (**C**) Waveform and statistical analysis of amplitude of iPSC-CMs; ** *p* < 0.01.

One day after microwave radiation, the IDs in group C were intact, and obvious gap junctions, tight junctions, and desmosomes were visible. Compared with group C, the ultrastructure of IDs in radiation groups became fuzzy and incomplete (Figure 7A), which might affect the electrical conduction function of iPSC-CMs.

#### 2.4.2. Electrical Conduction Dysfunction

The heart is one of the organs with abundant electrical activity. When the heart is damaged, the change in electrical conduction is also the most significant.

Through the detection of cell surface field potential, it was found that the conduction velocity of iPSC-CMs was slowed down and the conduction time was prolonged after microwave radiation, especially in the SX group (Figure 7B). In addition, the waveform showed that the amplitude of the cell beat decreased (Figure 7C). As a sensitive indicator, electrical conduction might be related to ID structure damage.

#### 2.4.3. Changes in Cx43 Expression in iPSC-CMs after Microwave Exposure

**Figure 8:**
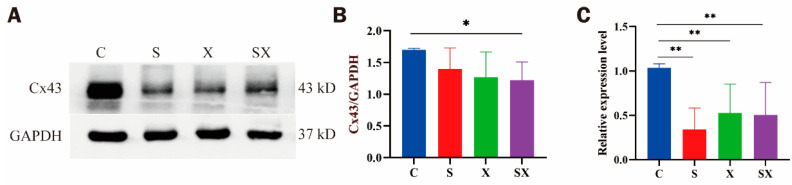
Changes in Cx43 expression in iPSC-CMs at 6 h after microwave exposure. (**A**) Protein bands. (**B**) Statistical graph of the OD ratio of the bands. (**C**) Relative mRNA expression of Cx43. * *p* < 0.05, ** *p* < 0.01.

Six hours after exposure, the results of Wb showed that compared with the C group, the expression of Cx43 in iPSC-CMs of the SX group decreased (*p* < 0.05, Figure 8A,B), and the expression of Cx43 in the S and X groups showed a downward trend; qRT-PCR results showed that compared with the C group, the relative expression of Cx43 mRNA in iPSC-CMs of the radiation groups was significantly decreased (*p* < 0.01, Figure 8C). This indicated that microwave radiation could reduce the expression of Cx43 in iPSC-CMs. Since hemichannels formed by Cx43 provide a direct conduit for ATP release, alterations of Cx43 hemichannels might be part of the mechanisms leading to microwave-radiation-induced mitochondrial dysfunction and cellular damage.

#### 2.4.4. Changes in Cx43 Distribution in iPSC-CMs after Microwave Exposure

**Figure 9:**
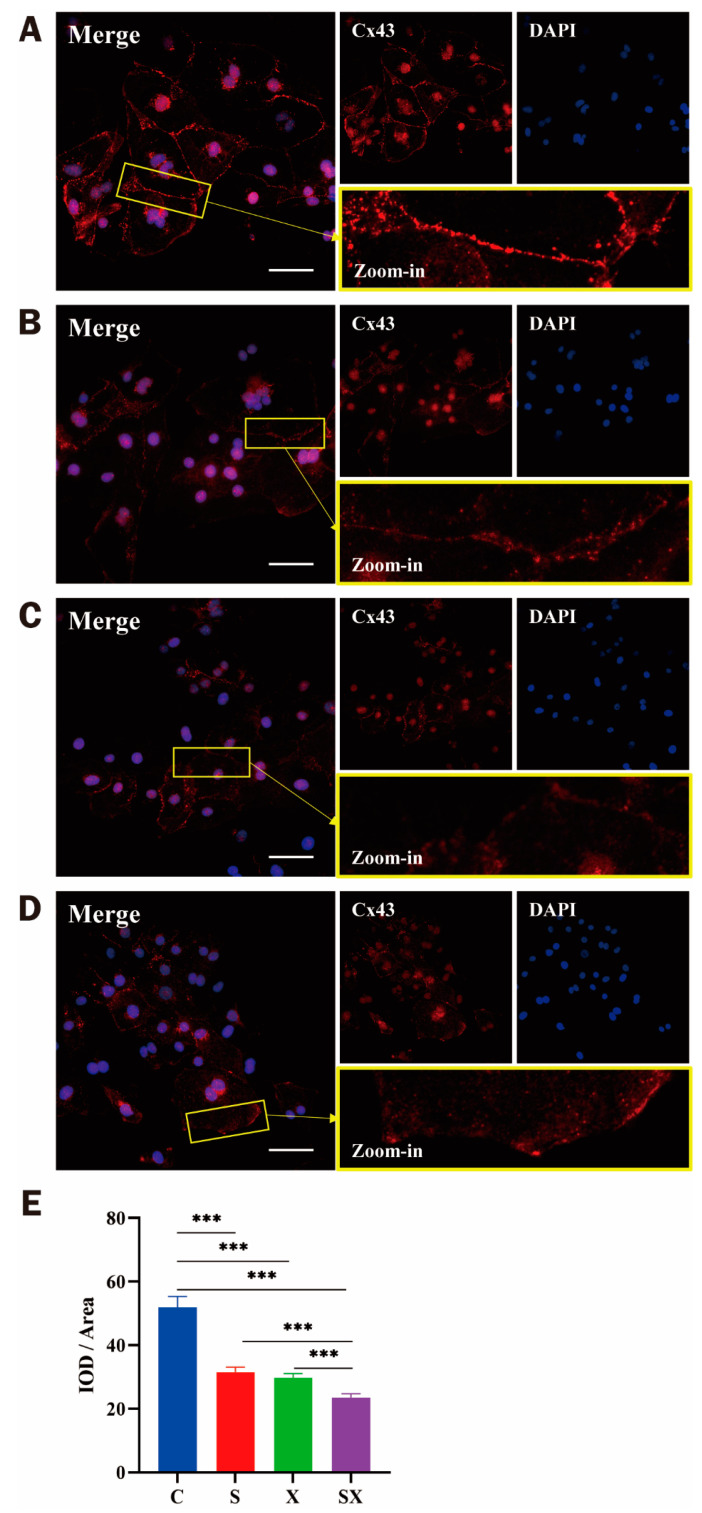
Changes in Cx43 distribution in iPSC-CMs at 6 h after microwave exposure. (**A**–**D**) Group C (control), group S, group X, and group SX, respectively; distribution of Cx43 in iPSC-CMs; scale bar = 100 μm; DAPI (blue), Cx43 (red). (**E**) Statistical analysis of the IOD/area ratio of the Cx43-positive signal. *** *p* < 0.001.

Six hours after exposure, the results of immunofluorescence staining showed that the positive signal of Cx43 at the cell–cell junction decreased in the radiation groups. After calculation and statistical analysis of the fluorescence intensity OD value, it was found that compared with the C group, the expression of Cx43 in the X group and the SX group was significantly decreased (*p* < 0.01); compared with the S group, the Cx43 expression in the X group and the SX group decreased (*p* < 0.05, Figure 9A–E). Based on the scattered structure of Cx43, it was speculated that chemical and electrical signal transmission disorders might be the key injury factors of myocardial cells induced by microwave radiation.

## 3. Discussion

Electromagnetic radiation has emerged as an important new source of pollution in modern civilization and has been classified as a possible human carcinogen by the International Agency for Research on Cancer [24,25], and its effects on the heart have also been demonstrated [26,27]. Because different microwave radiation source devices were not completely consistent, there was a lack of reproducibility in radiation conditions and methods of modeling. In the past decade, our research team has devoted a lot of time and effort to research on the biological effects of microwave radiation and has basically clarified the radiation dose and related damage effects that can cause injury in Wistar rats. Previous studies where Wistar rats were exposed to 30 mW/cm^2^ microwave radiation showed decreased heart rate, swollen cardiomyocytes, and damaged mitochondria [28,29]. A study by our group also found that there was a dose-dependent effect of microwave radiation on heart damage [30]. Although significant progress has been made in understanding radiation damage in Wistar rats, the translation of radiation dose from rats to humans is the subject of ongoing studies.

As one of the most important organs of life, the importance of the heart is self-evident. Cardiovascular disease is also an important culprit in human life, but the limited regenerative capacity of cardiac myocytes is the main reason for the high morbidity and mortality of various heart diseases. In recent years, with the development of medicine, cardiovascular treatment has been rapidly changing, from the transformation of surgery to internal medicine, which is transformed by large-scale surgery to minimally invasive surgery. At the same time, the close integration with regenerative medicine is also an important technological innovation for the future. An iPSC is a kind of totipotent stem cell with multi-directional differentiation potential that can proliferate infinitely in vitro and induce differentiation into cardiomyocytes. Due to the electrophysiological properties of iPSC-CMs with autonomous pulsation, they have been widely used in heart disease modeling and high-throughput drug screening [31,32]. In addition, some studies have shown that iPSC-CMs could be used to treat heart diseases such as myocardial infarction through the 3D culture of iPSC-CMs or by making cell patches, which has some advantages in improving local contractile function and energy metabolism [33,34]. The damage to the heart by microwave radiation is also manifested by the changes in the structure and function of the cardiomyocytes [35]. Introducing iPSC-CMs into the study of the effect and mechanism of microwave radiation on a cardiac injury can optimize the homology between the disease model and humans to a certain extent and solve the problem of the cell lines not having electrophysiological properties, which lays the foundation for further study of mechanisms and transformation of achievements.

Although iPSC-CMs have many advantages in this field, they still have some limitations compared with mature cardiomyocytes. An iPSC-CM is still considered an immature cell. We selected cells 7 days after recovery to develop a model that minimized variation in outcomes with cell status. By identification and observation of cell type, differentiation, and ultrastructure, we have found that the cells adhere to the dish uniformly, with clear morphology and regular pulsation; ultrastructure images showed that mitochondria in iPSC-CMs were abundant, and there were lamellar cristae mitochondria and tubular cristae mitochondria at the same time at 7 days after recovery [36,37]. The intercalated disc structure was visible, the myocardial fibers were abundant, and there were a large number of vesicle-like structures, indicating that the metabolism of cellular substances was active. Furthermore, the spontaneous pulsation frequency of cells was consistent with the human heart rate, which was the optimal state for experimental modeling.

After microwave radiation, the number of dead cells increased in the radiation group, and there was a dose-dependent effect. The detection was based on the membrane permeability of dead cells being greater than that of living cells. Furthermore, the content of myocardial enzymes in the cell culture medium was detected. Myocardial enzyme spectrum detection is an indispensable marker in the clinical screening of cardiac injury. As a protein stably expressed in the heart, cTnI is one of the must-check indicators when clinical myocardial infarction is suspected. H-FABP has been the most studied marker of early myocardial cell injury in recent years. Because of its high cardiac specificity and smaller molecular weight, it might be considered a more accurate indicator for the diagnosis of cardiac injury [38,39]. To explore the damaging effect of microwave radiation on iPSC-CMs, the ELISA method was used to detect the supernatant of the cell culture medium. We found that an increase in LDH and h-FABP appeared at 6 h after exposure, and the activities of AST, CK, and LDH increased at 1 d after exposure. The results showed that both myocardial enzymes and h-FABP could induce cell damage after exposure, and the increase in LDH and h-FABP appeared earlier than that of AST, CK, and cTnI. The increase in myocardial enzymes and damage markers further verified the damage to cell membrane structure and function by microwave radiation. Based on this speculation, we designed an experiment to observe the surface structure of the myocardial cell membrane, and we found that microwave radiation could indeed cause changes in the surface structure of the cell membrane, with different degrees of contraction and even rupture, and the integrity of the membrane structure was impaired. Microwave radiation could cause structural damage to the cell membrane. Another possibility is that the hemichannel structure was changed. After the hemichannels were affected by microwave radiation, the transmembrane transport of fluorescent dyes and myocardial enzymes was mediated by changing the open/closed state. Further studies are needed to discuss whether microwave radiation alters membrane permeability, hemichannel states, or both.

In addition to the cell membrane damage, the ultrastructure of other organelles was also observed, and it was found that the mitochondrial ultrastructure of iPSC-CMs was altered after radiation, showing an increased number of cavitated and ruptured mitochondria, and autophagosome-like bodies were found in mitochondria. This indicated that microwave radiation damages the mitochondrial structure in iPSC-CMs. The energy supply of the heart mainly comes from mitochondria, which are the main sites of myocardial energy metabolism and maintain normal shape and function through the dynamic balance of division and fusion [40]. The appearance of autophagosome-like bodies might be related to the self-repair after cardiac structural and functional disorders caused by the imbalance of mitochondrial power, which clears the damaged mitochondria, thereby maintaining the normal life activities of cells [41].

As the main energy-supplying organelles of cells, mitochondria are present in the heart in high amounts; in order to ensure the normal beating of the heart, they play the role of a power factory in the cell [42]. Therefore, we examined mitochondrial stress in cells after exposure and the response to mitochondrial respiration damage through basal respiration, proton leak, maximal respiration, spare respiration capacity, and ATP production. It was found that after microwave exposure, the mitochondria of the cells in the radiation group were damaged, which affected the energy metabolism and ATP generation of cells, thereby affecting their physiological processes. In addition to oxidative phosphorylation, glycolysis is also one of the major pathways for cellular energy production. We also detected the indicators of glycolysis in iPSC-CMs after radiation and found that glycolysis, glycolytic capacity, and glycolytic reserve decreased in radiation groups. The observation of alterations in the ultrastructure of mitochondria and of mitochondrial dysfunction demonstrates that the energy metabolism of iPSC-CMs was disturbed by microwave radiation. These results suggest that the structural changes of mitochondria, as the central energy producers of the cell, would inevitably lead to an impeded energy metabolism and loss of ATP generation, and these changes might be an important cause of abnormal electrophysiological function of cardiomyocytes.

In the course of observing the ultrastructure of iPSC-CMs, it was found that the ID structures were fuzzy after radiation. Based on the fact that structural damage could affect functional damage and the important role of ID in cardiomyocytes, the electrical conduction properties of the cells were studied, and the distribution of Cx43, a crucial player involved in conduction regulation, was analyzed. It was found that in the radiation groups, the conduction velocity decreased, the amplitude per beat decreased, and the expression of Cx43 related to conduction decreased. It was suggested that microwave radiation might affect the electrical conduction function of iPSC-CMs by reducing the expression of Cx43 in IDs.

Cellular communication is a form of cell-to-cell communication mediated by a family of connexins that can form intercellular channels, and it plays an important role in the maintenance of organ homeostasis in multicellular organisms. GJs are composed of two half-channels (connexons), consisting of six connexin proteins from each adjacent cell. Ions and intracellular messengers with molecular weights in the range of 1–1000 Da might be allowed to pass between connected cells via a gap junction or from the cytoplasm to the extracellular fluid via open hemichannels [43]. Cx43 is a major connexin in the heart and forms precursor hemichannels on cell membranes that accumulate at intercalated discs to assemble as GJs [44]. GJs not only play an important role in cardiac electrical conduction but also mediate the transfer of small molecules between adjacent cells. It has been shown that the ATP anion can regulate the proliferation of H9C2 through the Cx43 hemichannel [45], and Cx43 can also regulate the proliferation of H9C2 through the regulation of ATP and Ca^2+^ [46]. Studies have shown that extracellular ATP is mainly released through cell rupture or activation of hemichannels [47]. There is a Cx43-dependent ATP signaling pathway in macrophages, and by inhibiting this pathway, the secretion of inflammatory factors can be reduced, thereby improving the prognosis of sepsis [48].

In the condition of radiation damage, the cell barrier function is impaired, and hemichannels and gap junctions formed by Cx43 were found to be abnormally open, which can result in loss of ATP, ions, and other small molecules and the spreading of radiation damage signals between cells [49,50]. In this study, we found structural damage, dysregulation of energy metabolism, and impaired conduction function of iPSC-CMs, along with decreased expression and disordered distribution of Cx43 after microwave radiation. It has been inferred that microwave radiation might affect the structure and function of cells by affecting the expression and distribution of Cx43. It is further possible that changes in ATP or sodium and calcium ion content affect the cellular state linked with aberrant activity of Cx43 hemichannels, which mediates the transmembrane passage of small (<1.5 kDa) molecules. Future study of Cx43 proteins and associated channel activities after microwave radiation will provide further insight into the mechanisms of radiation damage and may lead to novel therapeutic strategies.

## 4. Materials and Methods

### 4.1. Cell Source and Culture Method

The iPSC-CMs (Cat. No. H01A00R18G, XYJ010001) were used in the experiment were purchased from Help Stem Cell Innovations (Nanjing, China). iPSC-CMs are also referred to as “cells” in the following text. The dishes were coated with 1% fibronectin for 1 h at 37 °C, and after counting with a cell counter (Countstar, Shanghai, China), the cells were seeded at 1 × 10^6^ cells/mL in 35 mm dishes (Corning, NY, USA) and cross-mixed to ensure that the cells were evenly plated. Cells for differentiation identification were seeded at 5 × 10^5^ cells/mL in laser confocal dishes with an inner diameter of 15 mm (801002, NEST, Beijing, China); cells for SEM and immunofluorescence were seeded at 2 × 10^4^ cells/100 μL on 14 mm round coverslips (YA0350, Solarbio, Beijing, China); cells for the mitochondrial stress and glycolysis stress test kit were seeded on 8-well assay plates (103025, Agilent, Santa Clara, CA, USA) at 3 × 10^4^ cells/80 μL. The recovery medium was changed to cell culture medium 24–48 h after plating and the medium was changed every 2 d thereafter.

### 4.2. Cell Identification

iPSC-CMs were stained with Human Cardiomyocyte Immunocytochemistry Kit (A25973, Invitrogen, Carlsbad, CA, USA) to verify the cell differentiation on the 7th d after recovery. The culture solution was extracted from the dishes first. The cells were incubated at room temperature for 15 min after being treated with fixative and permeabilization solutions, and then for 30 min after being treated with blocking solution. Then, the primary antibodies (Nkx2-5 for the early mesoderm and TNNT2/cTNT for the cardiomyocytes) were added directly to the blocking solution, mixed gently, and incubated at room temperature for 3 h. Then, 1× washing buffer was added 3 times and shaken for 3 min each time. The second antibodies were added and incubated at room temperature for 1 h. Then, 1× washing buffer was added 3 times and shaken for 3 min each time. In the last washing step, 2 drops/mL of NucBlue fixed cell stain were added and incubated for 5 min. Nkx2-5 (red), TNNT2 (green), and DAPI (blue) were observed under a CLSM (Nikon, Tokyo, Japan).

### 4.3. Groups and Exposure Methods

In this study, “S” and “X” represented 2.856 GHz and 9.375 GHz microwave exposure. The cells were divided into the following groups: C group (control group), S group (S-band microwave exposure group), X group (X-band microwave exposure group), and SX group (S- and X-band microwave composite exposure group).

Exposure methods: The radiation dose was 30 mW/cm^2^. The single exposure lasted 15 min, and the composite exposure group was radiated with the S-band first, and then with the X-band. Except for not receiving radiation, the treatment methods in group C were the same as those in other groups.

### 4.4. FCM

The cell viability was detected by FCM at 1 h after microwave radiation. The cell culture medium was discarded, and the cells were washed 3 times with 1 mL of DPBS (14190144, Gibco, Carlsbad, CA, USA); 1 mL of cell digestion solution was added (3005, Help, Nanjing, China), and the mixture was incubated for 5 min in a cell incubator. Cells were collected, 3 mL of stop solution was added (culture medium: FBS = 9: 1; 3001, Help, Nanjing, China; 12483020, Gibco, Carlsbad, CA, USA), and the mixture was centrifuged at 300 g for 5 min. The supernatant was discarded, 1 mL of DPBS was added to resuspend cells, and the mixture was centrifuged at 300× *g* for 5 min. Then, 1 mL of DPBS was added to resuspend cells, cells were counted, the density was adjusted to 1 × 10^6^ cells /mL, and 1 μL of fluorescent active dye solution (L23101, Invitrogen, Carlsbad, CA, USA) was added. After mixing, the cells were incubated at room temperature for 30 min under light, and the supernatant was discarded after centrifugation. After the cells were washed twice with 1 mL of DPBS containing 1%BSA (ST2249, Beyotime, Shanghai, China), the cells were resuspended with 1 mL of DPBS containing 1%BSA. Within 1 h after treatment, the cell viability was detected by FCM (BD, Franklin Lakes, NJ, USA).

### 4.5. ELISA

After exposure, the cell culture medium was taken and centrifuged at 10,000 r/min at 4 °C for 10 min, and ELISA (MEIMIAN, Yancheng, China) was used to detect the contents of cTnI, h-FABP, AST, CK, and LDH. Experimental methods: After blank wells (without sample and enzyme labeling reagents), standard wells (gradient dilution standard), and sample wells were set up, 50 μL of the standard substance was added to the standard wells, 40 μL of sample diluent and 10 μL of cell culture medium were added to the sample wells, and wells were incubated at 37 °C for 30 min after being sealed with a sealing film. After that, the samples were discarded, and washing buffer was added and let stand for 30 s; this was repeated 5 times, and wells were patted dry. Then, 50 μL of enzyme labeling reagent was added to each well, except for blank wells, and wells were incubated at 37 °C for 30 min after being sealed with a sealing film. After incubation, the liquid was discarded, followed by spin drying, the addition of washing buffer, standing for 30 s, washing 5 times, and patting dry. Then, 50 μL of chromogenic reagent A and 50 μL of chromogenic reagent B were added to each well, gently shaken, and mixed, and color developed at 37 °C for 10 min; then, 50 μL of stop solution was added to each well to stop the reaction. The blank well was set to 0, and the microplate reader was used to measure the absorbance of each well at 450 nm wavelength.

### 4.6. SEM

One day after exposure, the medium was discarded, and 2.5% glutaraldehyde fixative (pH = 7.2) was added, followed by incubation for 3 h at room temperature. This was followed by rinsing with phosphate buffer (pH = 7.2) 3 times, 10 min each time. Gradient dehydration was carried out with 50%, 70%, 80%, 90%, and 100% ethanol for 10 min each time (twice with 100% alcohol, and once with other concentrations). A carbon dioxide critical point dryer (EMITECH, South Petherton, UK) was used to dry samples, and an ion sputter coater (JEOL, Akishima, Japan) was used to spray gold coating. SEM (Zessi, Oberkochen, Germany) was used to observe and photograph samples.

### 4.7. TEM

One day after exposure, 1 mL of cell culture medium was discarded, and the cells were scraped off with a soft silica gel sheet, collected into a 1.5 mL EP tube, and centrifuged at 10,000 r/min for 10 min. After the supernatant was discarded, the cells were fixed in 2.5% glutaraldehyde fixative for 2 h and 1% osmic acid fixative for 1 h. Then, the cells were dehydrated with gradient ethanol and acetone transition, embedded in Epon812 resin, and made into ultrathin sections with a thickness of 70 nm, with lead–uranium staining. After these steps, TEM (Hitachi, Tokyo, Japan) was used to observe and photograph samples. After image acquisition, 5 visual fields were taken in each group, and the numbers of normal and abnormal mitochondria within 100 μm^2^ were counted and statistically analyzed.

### 4.8. Measurement of OCR and ECAR

OCR and ECAR were measured with the Seahorse XFp Extracellular Flux Analyzer (103010 and 103020, Agilent, Santa Clara, CA, USA). Cells were seeded on culture plates at 3 × 10^4^ cells/well and cultured at 37 °C for 2 d (the cells reached 90% confluence) before microwave exposure. The probe plates were hydrated 1 d before the measurement, and the experiment was carried out 1 h after radiation.

After exposure, the culture medium was changed to the basal medium (adding 25 mmol/L glucose and 1 mmol/L sodium pyruvate to the basal medium) before it was placed in a 37 °C incubator (without CO_2_) for 1 h. After monitoring baseline respiration, for OCR, 2 μM oligomycin, 1 μM FCCP, and 0.5 μM rotenone/antimycin A were automatically injected into each well. In order, 10 mM glucose, 2 μM oligomycin, and 50 μM 2-DG were used to measure ECAR. After each detection, the OCR and ECAR were analyzed using Wave software (Agilent, Santa Clara, CA, USA).

### 4.9. Electrical Mapping Recording

One hour after microwave radiation, the field potential of iPSC-CMs was detected using a multi-channel electrophysiological mapping system (MappingLab, Oxford, UK). After the cell medium was discarded, flexible MEAs (MappingLab, Oxford, UK) were gently attached to the surface of the cell membrane. The field potential was recorded using the EMapRecord5.7.7 system (MappingLab, Oxford, UK) for 30 s. After that, the conduction velocity and pulse amplitude were analyzed using the EMapScope5.8.0 system (MappingLab, Oxford, UK).

### 4.10. Wb

Total protein was extracted at 6 h after exposure, and the expression of Cx43 was semi-quantitated by Wb. The steps were as follows: After the cell culture medium was discarded, 1 mL DPBS (pre-cooled at 4 °C; 14190144, Gibco, Carlsbad, CA, USA) was added to the dish, followed by washing 3 times and discarding the liquid. Then, 100 μL RIPA (protease inhibitor:RIPA = 1:99; B14001, Bimake, Houston, TX, USA; P0013B, Beyotime, Shanghai, China) was added, followed by an ice bath for 10 min; a cell scraper was used to remove the cells, which were collected into 1.5 mL EP tubes, followed by an ice bath for 10 min; after centrifugation at 12,000 r/min 4 ℃ for 15 min, the supernatant was collected as total cell protein. The protein was mixed with the sample buffer (4:1; E151, Genstar, Beijing, China) and denatured in a boiling water bath for 10 min, and the cooled sample was subjected to the SDS-PAGE. After electrophoresis, membrane transfer, blocking, and cutting the PVDF membrane, rabbit anti-Cx43 (1:1000 dilution in TBST; ab11370, Abcam, Cambridge, UK) and mouse anti-GAPDH (1:10,000 dilution in TBST; ab8245, Abcam, Cambridge, UK) were added, and the bands were shaken at 4 °C overnight and washed with TBST 3 times, 10 min each time. Then, the HRP-labeled goat anti-rabbit IgG (diluted 1:10,000 in TBST; A0208, Beyotime, Shanghai, China) and goat anti-mouse IgG (diluted 1:10,000 in TBST; A0216, Beyotime, Shanghai, China) were added to each band, shaken at room temperature for 1 h, and washed with TBST 3 times, 10 min each time. The clear bands were used to semi-quantify the optical density values using a gel imaging system (CLiNX, Shanghai, China), and statistical analysis was conducted.

#### 4.11. qRT-PCR

Six hours after exposure, the mRNA expression of Cx43 was detected by qRT-PCR. After the total cell RNA was extracted using the Trizol method, the mRNA was reverse transcribed into a cDNA template with a Revertaid first-strand cDNA synthesis kit (K1622, Thermo Fisher Scientific, Waltham, MA, USA). The reaction system was as follows: 1 μL of Oligo (dT) 18 Primer, 4 μL of 5× Reaction Buffer, 1 μL of RiboLock RNase Inhibitor, 2 μL of 10 mM dNTP Mix, 1 μL of RevertAid RT, and 11 μL of total RNA and enzyme-free water. The reaction process was as follows: 60 min at 42 °C, 5 min at 70 °C, and then the termination of the reaction.

After the PCR reaction system was constructed with TB Green premix (RR820A, Takara, Nojihigashi, Japan), qRT-PCR experiments were performed using an Applied Biosystems 7300 Fast Real-Time PCR System (Applied Biosystem Technologies, Foster City, CA, USA). The reaction system was as follows: 10 μL of TB Green Premix Ex Taq II, 0.4 μL of ROX Reference Dye, 0.8 μL of forward primer, 0.8 μL of reverse primer, 2 μL of cDNA template, and 6 μL of enzyme-free water. The reaction process was as follows: pre-denaturation at 95 °C for 30 s, PCR reaction at 95 °C for 5 s, and 60 °C for 31 s (40 cycles). The primer sequences of Cx43 (GJA1) were as follows (5′ to 3′): forward primer sequence: AGTTCAATCACTTGGCGTGACTTC; reverse primer sequence: GTTTGCCTAAGGCGCTCCAG. The primer sequences of GAPDH were as follows (5′ to 3′), forward primer sequence: GCACCGTCAAGGCTGAGAAC; reverse primer sequence: TGGTGAAGACGCCAGTGGA.

### 4.12. Immunofluorescence

Six hours after exposure, the round coverslips were placed in a 24-well plate, and 1 mL of 4% paraformaldehyde (P0099, Beyotime, Shanghai, China) was added to each well for 15 min; 1 mL of permeabilization buffer with Trixton-X-100 (P0096, Beyotime, Shanghai, China) was added to rupture the membrane for 10 min; 1 mL of 10% goat serum (diluted 1:10 in PBS; C0265, Beyotime, Shanghai, China) was used as blocking solution at room temperature for 1 h. Then, 200 μL of Cx43 primary antibody (1:100 dilution in 10% goat serum; ab11370, Abcam, Cambridge, UK) was added and incubated for 1 h at room temperature; then, fluorescently labeled secondary antibody (1:100 dilution in 10% goat serum; ab150080, Abcam, Cambridge, UK) was added and incubated in the dark at room temperature for 1 h. The round coverslips were removed and put on slides, and after the DAPI mounting medium (H-1200, Vector Laboratories, Newark, CA, USA) was added, the square coverslips were covered and fixed with nail polish. The images were observed under a fluorescence microscope (Nikon, Tokyo, Japan). The optical density values were semi-quantified, and statistical analysis was conducted. The cells were washed with DPBS (14190144, Gibco, Carlsbad, CA, USA) 3 times, 3 min each time, before each medium change.

### 4.13. Statistical Analysis

The data in this paper are expressed as mean and standard deviation (x ± s), and SPSS 23.0 version was used for statistical analysis. Statistical analysis of differences between groups was performed using one-way ANOVA. The acceptable level of significance for all tests was *p* < 0.05, and the significant markers were as follows: * *p* < 0.05, ** *p* < 0.01, *** *p* < 0.001.

**Figure 10:**
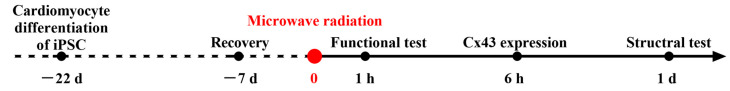
Timeline of experiments.

The experiment timeline is shown in Figure 10.

## 5. Conclusions

In this research, we used iPSC-CMs exposed to S- and X-band microwave radiation alone or in combination to simulate the damage to the heart caused by a special electromagnetic environment and further study the damage mechanism. We found that the cell membrane, mitochondria, and intercalated disc of iPSC-CMs were all damaged after microwave radiation, and the cell membrane permeability, cell energy metabolism, and electrical conduction functions were correspondingly affected. These abnormalities in structure and function were related to the decreased expression and disordered distribution of Cx43. Further studies are needed to elucidate the possible involvement of hemichannels in the cellular injury mechanism of microwave-radiation-induced damage.

## Data Availability

Not applicable.

## Abbreviations

μL: microliter; μm: micrometer; ANOVA: analysis of variance; AST: aspartate aminotransferase; ATP: adenosine triphosphate; cDNA: complementary deoxyribonucleic acid; CK: creatine kinase; CLSM: confocal laser scanning microscope; cm: centimeter; CO2: carbon dioxide; cTnI: cardiac troponin I; Cx43: Connexin43; d: day or days; DAPI: 4′,6-diamidino-2-phenylindole; DPBS: Dulbecco’s phosphate-buffered saline; ECAR: extracellular acidification rate; ELISA: enzyme-linked immunosorbent assay; EP tube: Eppendorf tube; ETC: electron transfer chain; FCCP: carbonyl cyanide p-trifluoromethoxy phenylhydrazone; FCM: flow cytometry; GAPDH: glyceraldehyde 3-phosphate dehydrogenase; GHz: gigahertz; h: hour or hours; h-FABP: heart fatty acid binding protein; HRP: horseradish peroxidase; IOD: integrated optical density; iPSC-CMs: cardiomyocytes derived from human induced pluripotent stem cells; LDH: lactate dehydrogenase; min: minute or minutes; mL: milliliter; mm: millimeter; mmol: millimole; mW: milliwatt; nm: nanometer; OCR: oxygen consumption rate; OD: optical density; PVDF: polyvinylidene fluoride; qRT-PCR: quantitative real-time polymerase chain reaction; RIPA: radio immunoprecipitation assay; RNA: ribonucleic acid; s: second or seconds; SDS-PAGE: sodium dodecyl sulfate-polyacrylamide gel electrophoresis; SEM: scanning electron microscopy; TBST: Tris-buffered saline with tween-20; TEM: transmission electron microscopy; TNNT2/cTnT: cardiac troponin T; Wb: Western blot.
